# Chimpanzees (*Pan troglodytes*) show subtle signs of uncertainty when choices are more difficult

**DOI:** 10.1016/j.cognition.2021.104766

**Published:** 2021-09

**Authors:** Matthias Allritz, Emma Suvi McEwen, Josep Call

**Affiliations:** a School of Psychology and Neuroscience, University of St Andrews, St. Andrews, Fife KY16 9JP, UK; b Department of Developmental and Comparative Psychology, Max Planck Institute for Evolutionary Anthropology, Deutscher Platz 6, Leipzig D-04103, Germany

**Keywords:** Chimpanzees, Feelings of uncertainty, Procedural metacognition, Transitive inference, Epistemic emotions

## Abstract

Humans can tell when they find a task difficult. Subtle uncertainty behaviors like changes in motor speed and muscle tension precede and affect these experiences. Theories of animal metacognition likewise stress the importance of endogenous signals of uncertainty as cues that motivate metacognitive behaviors. However, while researchers have investigated second-order behaviors like information seeking and declining difficult trials in nonhuman animals, they have devoted little attention to the behaviors that express the cognitive conflict that gives rise to such behaviors in the first place. Here we explored whether three chimpanzees would, like humans, show hand wavering more when faced with more difficult choices in a touch screen transitive inference task. While accuracy was very high across all conditions, all chimpanzees wavered more frequently in trials that were objectively more difficult, demonstrating a signature behavior which accompanies experiences of difficulty in humans. This lends plausibility to the idea that feelings of uncertainty, like other emotions, can be studied in nonhuman animals. We propose to routinely assess uncertainty behaviors to inform models of procedural metacognition in nonhuman animals.

## 1 Introduction

Humans can tell when they find a task difficult. Faced with a tough multiple-choice problem, we may catch ourselves as we are about to make a mistake, or we may notice that we are going back and forth between choices. Humans routinely judge the accuracy of their decision making and experience epistemic feelings like uncertainty, familiarity, or doubt. Philosophers have often mentioned that when humans report such feelings, this is accompanied by characteristic behaviors, e.g. wavering between options, hesitating, or frowning (Carruthers, 2017; Dokic, 2012; Proust, 2012).

This is consistent with the finding that explicit metacognitive appraisals (e.g. “this is very difficult for me”) are reliably associated with observable behavior. Rahnev et al. (2020) documented in an analysis of 4089 participants from 76 different datasets that when participants hesitate longer before giving an answer, they report lower confidence (mean *r* = −0.24). In recent years, new methods have been developed to identify the contributions that self-generated “uncertainty behaviors” make to metacognitive judgments. For example, Questienne, Atas, Burle, and Gevers (2018) studied the subjective experience of “urge-to-err”, an experience closely linked to perceived difficulty, in an arrow priming task. As in previous studies, experiencing a feeling of almost having made an error was correlated with response time. Crucially, this relationship was stronger when the response was preceded by a subtle EMG response from the incorrect hand than when it was not, implying that the metacognitive appraisal was sensitive to the experience of motor response competition. Similarly, Wokke, Achoui, and Cleeremans (2020) found that participants in a color discrimination task showed higher metacognitive sensitivity if they were asked about their confidence just after the presentation of a response cue (e.g. left button corresponds to green) than if they were asked before. In other words, the experience of a clear response tendency, or of competing response tendencies, contributed to how well participants could anticipate how likely they were to be correct. In another study, Dotan, Meyniel, and Dehaene (2018) presented participants with a touch screen task in which participants were asked to slide their finger either to the left or right in response to evidence that accumulated throughout the trial. Subtle changes in finger speed and acceleration suggested that online confidence monitoring affected the participants' speed. Post-decisional confidence ratings, in turn, correlated with the total variability in finger acceleration throughout the trial, and both were directly related to the objective difficulty of trials. In sum, evidence suggests that humans adjust their motor response preparation to their current level of confidence, and conversely, exploit their own experience of motor response competition in generating confidence and difficulty judgments.

Animal metacognition has been the topic of intense research for more than 20 years because of its conceptual overlap with philosophical questions about the subjective experience of agency in nonhumans (Metcalfe & Son, 2012), self-directed attention (Carruthers & Ritchie, 2012), and the evolution of theory of mind and self-other distinction (Carruthers, 2009; Musholt, 2015; Proust, 2007). The fact that humans appear to exploit self-generated motor responses when making metacognitive judgments is reminiscent of some of the earliest “cognitive conflict” models of metacognition in nonhuman animals (Smith et al., 1995). These mostly informal models posit that it is the experience of competing perceptual evidence or competing response tendencies without a clear “winner” that elicit “second-order” responses aimed at terminating this conflict (cf. Kepecs & Mainen, 2012). The majority of animal metacognition research focuses on these second-order behaviors that come to the rescue when a situation is too uncertain. These studies have found that different primate species (a) opt out of choices when trials are more difficult (Shields, Smith, & Washburn, 1997; Smith, Coutinho, Church, & Beran, 2013), (b) seek for information in a strategic and selective manner (Beran, Smith, & Perdue, 2013; Bohn, Allritz, Call, & Völter, 2017; Brady & Hampton, 2021; Call, 2010; Call & Carpenter, 2001; Kornell, Son, & Terrace, 2007; Malassis, Gheusi, & Fagot, 2015; Rosati & Santos, 2016), (c) and place larger “bets” on their own responses when they are more likely to be right (Kornell et al., 2007). Uncertainty behaviors, which signal a response conflict on the level of the primary response, have received much less empirical scrutiny on the other hand, even though they have occasionally been described (Muenziger, 1938; Smith et al., 1995; Tolman, 1926).^1^

Uncertainty behaviors have implications for two overlapping debates. The first concerns if and how animals experience epistemic feelings. The premise that animals experience “feelings of uncertainty” is acceptable to some (e.g. Carruthers & Ritchie, 2012; Proust, 2012), while others remain non-committal or skeptical (e.g. Hampton, 2009; Smith, Shields, & Washburn, 2003). Rather than relying on philosophical stances, we propose that it may be possible to study epistemic emotions with similar methods as they have in recent years been developed for studying other emotions in nonhuman animals (Mendl, Burman, & Paul, 2010; Panksepp, 2011; Paul, Harding, & Mendl, 2005). For example, some have proposed that careful examination of the componential structure of responses to specific situations (e.g. motor behavior, physiological responses, cognitive biases) may allow us to determine to what extent experiencing a specific emotion is distinct from other emotional experiences in the same species (Paul et al., 2005). Moreover, by comparing multiple components across different situations, we can speculate to what extent the subjective experience of uncertainty is isomorphic between humans and other species (Panksepp, 2011). Documenting wavering in nonhuman animals in studies that are analogous to the ones recently conducted with humans, could thus be a first step in assembling what “feeling uncertain” looks like in nonhuman animals.^2^ Triangulating epistemic feelings in this way is more than a mere philosophical exercise, because it is these feelings that are presumed by some to give rise to the higher-order, conflict-resolving behaviors (e.g. seeking information, opting out) that one may regard as metacognitive (Dokic, 2012; Proust, 2012).

Second, irrespective of any assumptions one may have about the involvement of subjective feelings, whether animals show uncertainty behaviors has direct implications for evaluating models of “procedural metacognition” (Beran, Brandl, Perner, & Proust, 2012; Hampton, 2009). Procedural (or implicit) metacognition refers to the monitoring and control of cognitive processes and abilities (Proust, 2019). This allows animals to predict and improve the results of their thinking via simple heuristics like “if it cannot be remembered easily, then seek more information”. The term “procedural metacognition” is typically used to differentiate the concept from “declarative” or “metarepresentational” forms that, by definition, require that these heuristics must themselves be explicitly represented. A demonstration regarded necessary for either type of metacognition, procedural and declarative, is that the cues that give rise to metacognitive behaviors in the first place are “endogenously-generated” or “private” (Beran et al., 2012; Hampton, 2009). Uncertainty behaviors like wavering are particularly interesting in this context because they involve both proprioception (a purely endogenous cue) and movement (what Hampton, 2009, would call a “publicly available cue”). How should these behaviors be treated in animal metacognition experiments, then?

On the one hand, the recent studies with humans show that uncertainty behaviors without a doubt contribute to reporting of appraisals that are conventionally considered metacognitive in humans, like confidence or difficulty judgments (Dotan et al., 2018; Questienne et al., 2018; Wokke et al., 2020). On the other hand, some researchers of animal metacognition caution that self-generated cues, if they are publicly available like motor behavior, may trigger conditioned responses (e.g. choosing an opt-out button) that only look like metacognitive behaviors, but may just as well be described as reward-maximizing, learned response chains (Hampton, 2003, Hampton, 2009; Proust, 2012). For the most convincing demonstrations, we may thus need to exclude this possibility via statistical control (Goupil, Romand-Monnier, & Kouider, 2016) or experimental design (Basile, Schroeder, Brown, Templer, & Hampton, 2015; Kornell et al., 2007). For the most fair comparison, on the other hand, one might argue that if animals respond to their self-generated behavioral cues with adaptive second-order behaviors, there is no good reason not to call this metacognitive as well (cf. Kornell, 2014, for a related discussion). Regardless of where one stands on this issue, it follows that documenting uncertainty behaviors routinely is critical for refining existing theories of animal metacognition.

In spite of their importance in the debates over epistemic feelings and procedural metacognition, wavering and similar subtle, observable signs of response competition have been studied only rarely. In the classic study by Smith et al. (1995), a bottlenosed dolphin was trained to press one paddle in response to high-pitched tones, one paddle in response to low-pitched tones, and a third paddle to escape an ongoing trial in exchange for an easier one. In addition to using this uncertainty response more often as a function of pitch ambiguity, Smith et al. reported that in some trials the dolphin wavered between the primary options. The amount of wavering was distributed like the choice of the escape response along the pitch continuum, and wavering often foreshadowed an escape. The authors interpreted these behaviors as expressions of cognitive conflict at discrimination threshold, which in turn “elicit[s] higher modes of cognition”. Sayers, Evans, Menzel, Smith, and Beran (2015) described a similar phenomenon in the rhesus monkey Murph, who completed a sparse-dense discrimination task and who showed more joystick wavering in those trials in which he eventually chose to opt out. Two other studies have investigated the relationship between “hesitation” and task performance in great apes (Suda & Call, 2006) and in captive fur seals (Scheumann & Call, 2004). However, relating these findings to studies of wavering and metacognition in humans is complicated by the facts that hesitation scores in both studies collapsed rather different types of motor behaviors, and that the relationship between hesitation and difficulty was based on subjects' aggregated performances on the level of individuals or test conditions, respectively. For example, Suda and Call (2006) investigated whether great apes' hesitation was related to performance in a Piagetian liquid conservation task. Wavering back and forth between options, and the simultaneous picking of two options with both hands, were both collapsed in a single measure of “hesitation”. While the former behavior is very similar to the oscillating motor responses shown in human EMG and touchscreen studies (Dotan et al., 2018; Questienne et al., 2018), using both hands simultaneously may not necessarily be a manifestation of decisional or response conflict. It could, for example, reflect an incomplete understanding of the task requirements by some subjects (i.e. that only a single choice is allowed per trial), or it may have served as an acquired, second order behavior, used by subjects to move the experiment along, akin to primates using the “opt-out” option in other tasks.

Second, unlike in human studies, hesitation was not investigated with regard to how it changed as a direct consequence of trial-to-trial variations in objective difficulty. Rather, subject averages in hesitation were correlated with averages in performance. An inverse U-shaped relationship best accounted for the data. The authors regarded this as evidence that those subjects who showed an intermediate performance must have experienced most strongly a conflict between two different problem-solving strategies, and consequently showed hesitation most often. While this may indeed be the case, correlations of averages alone cannot explain what makes subjects hesitate more in some trials than in others. In sum, collapsing different types of “hesitation” into one measure, and correlating them with performance averages both complicate the comparability with human studies of uncertainty behaviors (Dotan et al., 2018; Questienne et al., 2018; Wokke et al., 2020), and thus inferences about epistemic feelings and metacognitive appraisals. A task for nonhuman primates that can be compared directly with human studies of uncertainty behaviors requires an unambiguous, quantitative measure of wavering, compared across test conditions of varying degrees of objective difficulty.

To fill this gap, we explored uncertainty behaviors in a touchscreen task with three chimpanzees at the Wolfgang Koehler Primate Research Center (WKPRC) in Leipzig Zoo, Germany. Several studies have demonstrated metacognitive behaviors in response to experiencing uncertain situations in chimpanzees. For example, chimpanzees have been shown to seek for information selectively when required to locate hidden food (Call, 2010; Perdue, Evans, & Beran, 2018) or the best tool (Bohn et al., 2017), or to identify a hidden food (Beran et al., 2013); and they are more likely to move and collect a reward after responding correctly, even before receiving performance feedback (Beran et al., 2015). Less is known, however, about chimpanzees' experience of uncertainty itself and whether this experience can be quantified. Here, we investigated whether the three chimpanzees would show more hand wavering between two pictures on a screen whenever they were presented with more difficult choices. Rather than training our chimpanzees in one of the established metacognition tasks (e.g. opt-out or betting paradigms), we collected wavering data in the context of an already ongoing study on serial learning and transitive inference.^3^ This choice allowed greater comparability with human findings on uncertainty behaviors because for transitive inference tasks, objective, gradual differences in difficulty of individual probe trials are well established based on a large body of literature. To further approximate the fine-grained measurement of human EMG and touchscreen studies, we recorded the amount of wavering not as a binary variable but as a count, allowing us to relate gradual differences in trial difficulty to gradual differences in overt wavering for each individual directly.

Transitive inferences are inferences of the type “if A > B and B > C, then A > C”. In comparative studies, relationships of this type are typically operationalized in terms of sequential order, e.g. “press A before you press B", etc. To learn an implied list (e.g. A > B > C > D > E), subjects (often pigeons or rhesus macaques) are initially trained to make correct selections for all premise pairs (“AB”, “BC”, “CD”, “DE”, for reviews, see Jensen, 2017; Vasconcelos, 2008), though in some cases subjects are initially trained on the full list (“ABCDE”, see e.g. Jensen, Altschul, Danly, & Terrace, 2013; Templer, Gazes, & Hampton, 2019). Training is followed by probe trials in which subjects are presented with all possible item pairs (e.g. “BD”). Contemporary studies often focus on the cognitive representation of the distance between list items (Gazes, Lazareva, Bergene, & Hampton, 2014; Jensen, Muñoz, Alkan, Ferrera, & Terrace, 2015; Lazareva, Paxton Gazes, Elkins, & Hampton, 2020; Templer et al., 2019). One result that stands out is the so-called symbolic distance effect: newly trained subjects find it easier to pick the item that comes first when the distance between two items along the implied list is large (e.g. picking B over E vs. picking B over C). They respond more accurately, more quickly, or both. This effect has been found repeatedly in multiple primate species (Jensen, 2017), both after traditional premise pair training (e.g. Merritt & Terrace, 2011) and when training involved learning the full sequence (e.g. Jensen et al., 2013). A second, related effect is the magnitude effect (Terrace, 2012), also sometimes called the first item effect: performance is generally better, the closer the first of the two subset items is to the beginning of the list (e.g. picking A over C vs. picking B over D). This effect has also been demonstrated multiple times (e.g. Templer et al., 2019; Terrace, Son, & Brannon, 2003).

We used symbolic distance and magnitude as a proxy for task difficulty in our study of wavering. To train our three chimpanzees on an implied list of five items, we used a serial learning task with an increasing number of images present (training A-B-C, then A-B-C-D, and finally A-B-C-D-E). After successful training, we introduced probe trials that presented subjects with all potential item pairs (AB, AC, …, DE). We investigated how wavering was affected by magnitude and symbolic distance, because, based on a large body of published research, these two dimensions represent objective and highly replicable correlates of trial difficulty. Crucially, we expected that wavering between items, if it occurred, should show comparable patterns, occurring more frequently when item pair magnitude was larger, and when symbolic distance was smaller. Demonstrating this relationship would achieve two things. First, it would constitute a behavioral analogue to human uncertainty behaviors that precede verbal reports of experiencing difficulty and low confidence (Questienne et al., 2018; Wokke et al., 2020). Though not conclusive evidence in and of its own, this would lend plausibility to the idea that some animals feel uncertainty in a way similar to humans. Second, it would suggest that response conflict can easily be operationalized and quantified non-invasively in tasks that present a simple manual choice. Routinely incorporating such measurements in animal metacognition studies would allow us to refine and expand current models of procedural metacognition (Carruthers & Ritchie, 2012; Hampton, 2009; Proust, 2012). In addition to the relationships between difficulty and wavering, we expected to replicate the typical magnitude and distance effects with regard to response latency or accuracy, or both, with the chimpanzees in this study.

## 2 Method

### 2.1 Subjects

Three chimpanzees participated in the study: male Alex (13 years at the time the wavering data was collected), female Jahaga (22 years), and male Kofi (9 years). Training with the serial learning task was also attempted with two additional subjects (female Sandra, 21 years, and male Lome, 13 years) but was not completed to the last stage because the subject either lost interest in the test or because of time constraints. All subjects had experience with regular touchscreen tasks for a year or more before data collection for this study began, and this included a serial learning task, a transitive inference task and a memory task involving Arabic numerals for two of the subjects (Alex and Jahaga), and a transitive inference task for one of the subjects (male Kofi).

Chimpanzees were housed at the WKPRC in Leipzig Zoo, Germany where they lived in a social setting in an indoor enclosure containing climbing structures and foraging boxes for enrichment purposes, with seasonal access to an outdoor enclosure. Their diet consisted of vegetables, fruit, and occasional meat and eggs. Subjects also received enrichment food items to encourage foraging behavior. In the morning of each testing day, access was made available to a testing room and subjects were given the option to enter and participate in cognitive tasks and earn food rewards, additional to their regular diet. Participation was entirely voluntary and non-invasive, and subjects were never food or water deprived. Water was available at all times, both in the enclosures and testing rooms. Individuals were separated for testing, other than from dependent offspring. All research and husbandry complied with the European Association of Zoos and Aquaria (EAZA) and the World Association of Zoos and Aquariums (WAZA) regulations. All research was also approved by the responsible committee at the WKPRC which at the time consisted of the director of WKPRC, the research coordinator, the head keeper and assistant head keeper of great ape husbandry, and the zoo veterinarian.

### 2.2 Apparatus

The setup is described in detail in Allritz, Call, and Borkenau (2016). Chimpanzees were presented with a transparent infrared touch screen mounted in front of a 19 in monitor (aspect ratio 5:4, resolution 1280 × 1024 px). All experimental programs were created with *E*-Prime version 2.0.8.90 running on a Windows 7. The touchscreen was in a fixed position, and always in the same location for the same subject. At the beginning of the session, the screen was blocked by a plastic panel. Once the subject entered the testing room and was in front of the screen, the panel was removed, and the screen revealed. For each correct response, a piece of apple or grape was handed to the subject by the experimenter. If subjects did not respond for approximately 10 min or showed any signs of distress, testing was terminated. All sessions were video recorded.

### 2.3 Training: Trial procedure

**Fig. 1:**
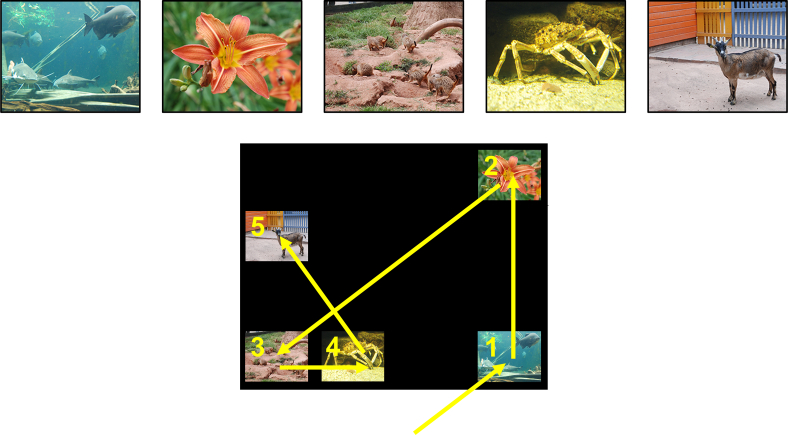
Top: the five image stimuli used as list items. Bottom: example trial with required clearing order. For details, see text.

The goal of training was for subjects to learn to clear five items (color images) off a touch screen in the correct order. All stimuli were presented on a black background for subjects Alex and Jahaga, and on a white background for subject Kofi. Different color backgrounds were used for different subjects in preparation of a different, unrelated set of experiments that was conducted after this study was completed. The items used were 260 × 208 px color bitmap files (see Fig. 1), thus, on the touch screen they appeared at a size of ca. 7.7 by 6.1 cm.

Fig. 1 depicts an example trial. Each trial in the training stages began with the presentation of a central initiation symbol. Upon touching this symbol, depending on the training stage, the first two, three, four, or all five items that were part of the list appeared on the screen. Each item appeared in one of 16 locations on a virtual 4 × 4 grid of possible locations on the screen. Within a trial, each correct touch was followed by the disappearance of the touched item and a chime (“Windows XP Default.wav”), which subjects had already learnt to associate with correct performance in previous touch screen tasks. If subjects touched all items in the correct order, they received a food reward (a piece of apple or grape), and the initiation symbol for the next trial appeared after 750 ms. If a subject touched any of the presented items too early, all remaining items simultaneously disappeared, followed by a timeout of 2000 ms and the subject receiving no reward.

### 2.4 Training: Session procedure

Training sessions were conducted opportunistically and subjects were able to complete up to 100 training trials per day. If a subject stopped participating, testing was terminated and the remainder of the 100 trials scheduled for that day were completed on the next available testing day. Incomplete sessions of this type occurred rarely (3 out of 361 training sessions across all three subjects). Data from trials that were abandoned mid-trial were discarded, and these trials were repeated when the session was completed. Subject performance was evaluated after each completed session of 100 trials to determine whether the subject should be promoted to the next training stage.

### 2.5 Training: Schedule

All subjects completed a stepwise procedure, learning e.g. to complete a list of the first three items, then a list of the first four items, and finally the list of all five items in the correct order. Each time a subject completed two consecutive sessions of a training stage with at least 81% of trials correct in each, they were promoted to the next training stage, and upon completing the final training stage in this manner, they were promoted to the test. One subject was not able to reach this criterion within 100 sessions during their four-item and five-item training and was instead promoted to the next stage directly after having completed 100 sessions of the respective training stage (see Table 1). Subjects Alex and Jahaga started training with the three-item list, whereas subject Kofi started training with a two-item list. This was done because Alex and Jahaga already had experience with a different serial learning task. Table 1 gives a summary of training conditions and progress. Fig. S1 provides a full overview of the subjects' training progress over time.

**Table 1:** Training sessions to criterion in serial learning task.

Subject	Sex	Age	Training Condition	Sessions to Criterion	Performance in final two sessions (correct trials)
Alex	m	13	3 item list	8	86, 86
			4 item list	12	81, 86
			5 item list	33	86, 85
Jahaga	f	22	3 item list	7	83, 82
			4 item list	100⁎	80, 69
			5 item list	100⁎	68, 72
Kofi	m	9	2 item list	4	99, 97
			3 item list	16	81, 81
			4 item list	38	86, 88
			5 item list	43	84, 83

⁎ Subject did not reach criterion of two consecutive sessions with performance of at least 81% and was promoted to next stage after 100 completed sessions instead.

### 2.6 Test: Transitive inference

Each subject completed eight test sessions of 100 trials each. In each session, 70 “regular trials” were identical to training trials from the last training condition, presenting subjects with all five items. On the remaining 30 trials (“subset trials”), the subject was presented with one of the ten possible unique two-item subsets from the implied list (subsets 1–2, 1–3, 1–4, 1–5, 2–3, 2–4, 2–5, 3–4, 3–5, 4–5). The presentation of regular trials and test trials within a test session was completely randomized across subjects and sessions. Responding on subset trials was non-differentially reinforced to prevent learning about subsets from feedback (Vasconcelos, 2008). This means that if a subject touched first the latter of the two items in a subset trial, it disappeared and a chime was played as if the subject had made a correct choice, leaving the earlier item to be cleared second. Each of the ten unique subset trials was presented three times per test session. Thus, across the eight sessions, each unique pair was presented 24 times, resulting in a total sample of 240 subset trials per subject. As in all other trials, the positions in which the two items in subset trials appeared were selected randomly before each trial. Because spatial distance between items may contribute to the degree with which wavering could be detected, we tested for relationships between spatial distance and our two main predictors, symbolic distance and magnitude (for definitions, see below). We calculated Pearson correlations between the spatial distance between items (measured in pixels from item center to center) and subset trial magnitude, across all trials for which wavering data was also available. These correlations were very small and not significantly different from 0 for all three subjects (Alex: *r*(238) = −0.04, *p* = .575; Jahaga: *r*(208) = 0.08, *p* = .237; Kofi: *r*(238) = −0.02, *p* = .787). Correlations between spatial distance and symbolic distance were similarly small for all subjects and significantly different from 0 only in one case (Jahaga: *r*(208) = −0.14, *p* = .045; Alex: *r*(238) = 0.01, *p* = .897; Kofi: *r*(238) = 0.04, *p* = .584). A sensitivity test confirmed that including vs. excluding spatial distance as an additional predictor made no difference to the statistical inference regarding the effect that symbolic distance had on Jahaga's wavering.

### 2.7 Behavior coding

All subset trials were coded by one of the authors (EM) for instances of overt wavering (see Table 2), that is spontaneous deviations from a seemingly set course towards one of the items, either towards the other item or to another location on the screen. Specifically, we coded all instances during a subset trial in which the subject's hand paused (*Rest*, Table 2) or changed (*Turn*, Table 2) direction before selecting an item, rather than moving directly to and immediately touching it.

**Table 2:** Wavering behavior coding scheme.

Action	Definition
*Move to*	The subject moves their hand from a resting, turning or touching location to another resting, turning, or touching location.
*Touch*	The subject touches the screen at the position of an item (or very close to) with tip of their finger, thumb, or knuckle.
*Turn*	The subject's hand changes direction, either while above or on the way to an item, or back towards an item it has just moved away from.
*Rest*	The subject's hand hovers over a stimulus without touching it.

In all analyses that follow, “wavering” refers to the total count of turns and rests that occurred before the first item was touched, as coded by EM. All behavior coding was carried out with Mangold INTERACT software, which allows viewing and time-stamping on the level of individual video frames (videos had a frame rate of 25fps). Critical areas and minimum durations were defined in a more detailed version of this coding scheme to help coders decide what constituted e.g. a Turn or a Rest in borderline cases, and to achieve satisfactory interobserver reliability (see Supplementary Materials). For examples of wavering, see Supplementary Video SV1.

The first observer coded all but one of the 24 sessions that the three chimpanzees completed in total. One session could not be coded for wavering because no video was recorded due to experimenter error. Of the available 23 video sessions, three were chosen from each subject to be coded by a second coder, yielding 270 of the total 690 trials (39.13%) as the reliability sample. Interrater reliability of the wavering count per trial was moderate to good by common conventions (Pearson *r*(268) = 0.72, ICC(2) = 0.70, see Cicchetti, 1994) and similar to reliability estimates for other reported count measures that include subtle animal movements, e.g. frequency of gaze alternation in canines (Marshall-Pescini, Rao, Virányi, & Range, 2017) or motor action diversity in birds (Logan, 2016).

### 2.8 Data analysis

#### 2.8.1 Wavering

We fitted GLMMs with Poisson error distribution and log-link function (R package lme4, function glmer, see Bates, Mächler, Bolker, & Walker, 2014), predicting the count of wavering behaviors per trial as a function of either magnitude or distance. Symbolic distance (ranging from 1 to 4) was defined as the difference between the list positions of the two subset items. Magnitude (also ranging from 1 to 4) corresponded to the position of the smaller of the two subset items in the original five-item list. Symbolic distance and magnitude entered each statistical model as a continuous, rather than nominal or ordinal predictor, consistent with multiple studies of transitive inference and serial learning in nonhuman primates that have shown that subjects cognitively represent items along a linear spatial continuum (Gazes et al., 2014; Jensen et al., 2013). In addition to each main predictor, “trial” (counting trials across all eight sessions from 1 to 24 for each of the 10 subset pairs) was included as predictor to control for any learning effects across subset trials. Statistical significance of the distance or magnitude effect was determined via likelihood ratio test, comparing this full model with one that was identical except for the critical predictor, using the drop1 function of the R package lme4. Each model also included stimulus pair as random effect with a random intercept term. Wavering models did not include a random slope term (which would estimate the variation of trial effects across stimulus pairs). Though including a random slopes term resulted in similar or identical fixed effects parameter estimates for magnitude and distance for all models, it sometimes resulted in singular model fits. Because statistical inference via likelihood ratio tests is not recommended for models with singular fit (Bates et al., 2020), results are reported for models that only include a random intercept term. Assumption checks did not indicate overdispersion to be an issue with any of the models, dispersion parameters, using the formula suggested by Bolker (2021), were 0.96, 0.83 and 0.78 for the magnitude models for Alex, Jahaga and Kofi, respectively, and 0.92, 0.86, and 0.73 for the distance models. Consistent with findings in humans that have established a relationship between behavioral signs of uncertainty and reported task difficulty, we predicted that the amount of wavering in chimpanzees would reflect task difficulty as well: trials with higher magnitude and smaller symbolic distance should be accompanied by more wavering.

#### 2.8.2 Latency

To assess whether magnitude and distance effects in our study replicated those frequently reported in the literature, we fitted two Linear Mixed Models with Gaussian error distribution (R package lme4, function lmer). Both models predicted log-transformed latency to touch the first item within a given trial as a function of either magnitude or distance, and trial number. In addition, “pair” (the specific subset, e.g. “1–4”) was entered as a random effect with a random intercept term. Similar to the models of wavering, latency models that also included a random slope term for the interaction of pair and trial converged on nearly identical fixed effects parameter estimates but in some cases resulted in singular fits. Thus, only the results of models with a random intercept (but without random slopes) are reported.

#### 2.8.3 Accuracy

We fitted GLMMs with binomial error distribution and logit-link function (R package lme4, function glmer), predicting whether items in a trial were cleared in correct order or not as a function of either magnitude or distance. Again, “trial” was included in all models as an additional predictor to control for learning effects. All but one model also included stimulus pair as random effect with a random intercept term. Models did not include a random slope term because, when included, these models or their respective null models sometimes resulted in singular fits (see above). For one subject (Kofi), the random intercept model for a magnitude effect on accuracy also resulted in a singular fit. For this case, results are reported for a simple logistic regression (function glm) that included only fixed effect terms. Inclusion or exclusion of random effects terms did not affect statistical inference for any of the models (comparing *p*-values implied by likelihood ratio tests with the conventional alpha level of 0.05). An exploratory analysis of trial effects on accuracy, latency and wavering can be found in the Supplementary Materials. The data collected for this study can be accessed at osf.io/g64ms.

## 3 Results

### 3.1 Wavering

Wavering occurred at least once in 227 (32.9%) of the 690 coded subset trials and ranged between 1 and 4 wavering movements in these trials. Between the three chimpanzees, the mean number of wavering movements across all subset trials ranged from 0.35 to 0.54 (Alex: *N* = 240, *M* = 0.54, *SD* = 0.80; Jahaga: *N* = 210, *M* = 0.35, *SD* = 0.67; Kofi: *N* = 240, *M* = 0.45, *SD* = 0.75). Predictably, the number of wavering movements correlated substantially with log-transformed response latency (Alex: *r*(238) = 0.61; Jahaga: *r*(208) = 0.63; Kofi: *r*(238) = 0.71; all *p* < .001). A full breakdown of wavering movements per subject per item pair can be found in Fig. S2a and S2b (Supplementary Materials).

**Fig. 2:**
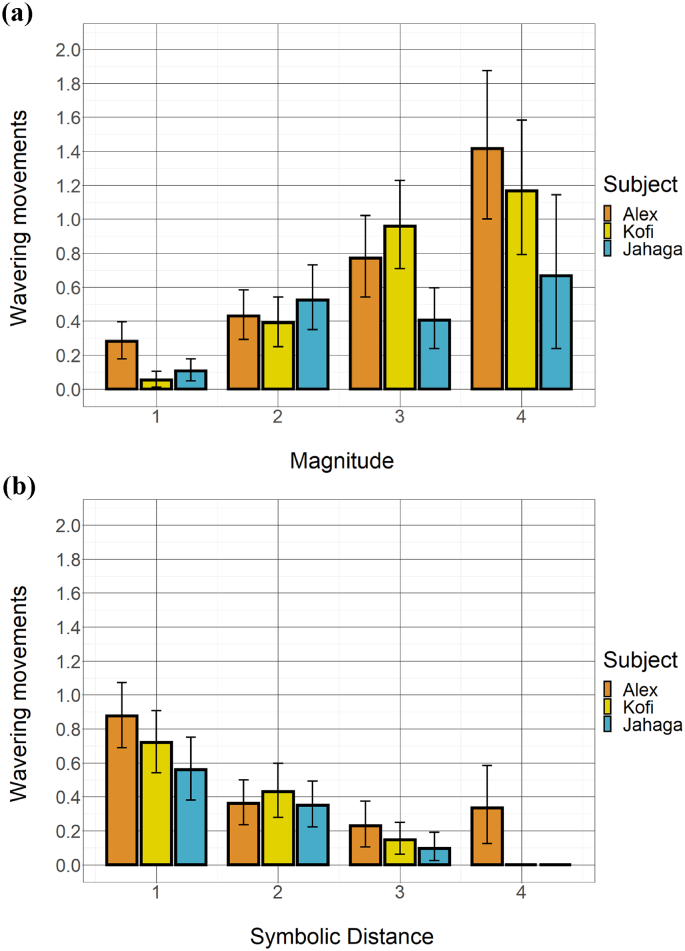
Effects of (a) magnitude and (b) symbolic distance of subset pairs on chimpanzees' number of wavering movements throughout the trial. Error bars represent confidence intervals (nonparametric bootstrap).

Fig. 2a depicts the number of wavering movements as a function of magnitude. Fig. 2b depicts the number of wavering movements as a function of symbolic distance. Overall, all three chimpanzees wavered more with larger subset magnitude and with smaller symbolic distance. These differences were statistically significant for all comparisons for the three subjects (Magnitude, Alex: β = 0.54, Χ^2^(1) = 11.22, *p* = .001; Jahaga: β = 0.67, Χ^2^(1) = 5.48, *p* = .019; Kofi: β = 1.02, Χ^2^(1) = 16.56, *p* < .001, Distance, Alex: β = −0.46, Χ^2^(1) = 5.35, *p* = .021; Jahaga: β = −0.93, Χ^2^(1) = 11.01, *p* = .001; Kofi: β = −0.95, Χ^2^(1) = 5.72, *p* = .017). A comparison of model predictions and empirical data can be found in Fig. S5a and S5b. For examples of wavering movements, see supplementary video SV1.

### 3.2 Latency

**Fig. 3:**
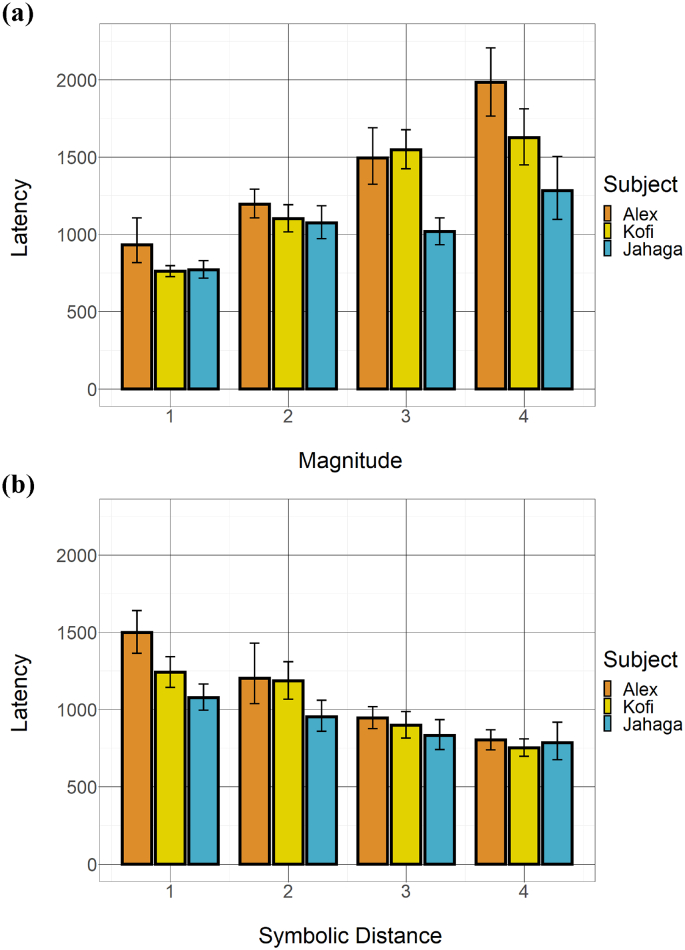
Effects of (a) magnitude and (b) symbolic distance of subset pairs on chimpanzees' latency to touch the first of the two list items. Error bars represent confidence intervals (nonparametric bootstrap).

Median response latencies for clearing the first item across all subset trials ranged from 828 ms to 1026.5 ms between subjects (Alex: *Mdn* = 1026.5, *M* = 1228.10, SD = 703.82; Jahaga: *Mdn* = 828.0, *M* = 961.53, *SD* = 414.17; Kofi: *Mdn* = 932.50, *M* = 1106.13, *SD* = 481.07). Fig. 3a depicts response latency as a function of magnitude of the smaller subset item. Fig. 3b depicts response latency as a function of symbolic distance between the two subset items. A full breakdown of response latency per subject per item pair can be found in Fig. S3a and S3b (Supplementary Materials). With very few exceptions, the three chimpanzees responded more slowly with larger subset magnitude and with smaller symbolic distance. These differences were statistically significant for most comparisons for the three subjects (Magnitude, Alex: β = 0.26, Χ^2^(1) = 22.77, *p* < .001; Jahaga: β = 0.16, Χ^2^(1) = 12.49, *p* < .001; Kofi: β = 0.29, Χ^2^(1) = 27.04, *p* < .001, Distance, Alex: β = −0.19, Χ^2^(1) = 6.20, *p* = .013; Jahaga: β = −0.11, Χ^2^(1) = 4.71, *p* = .030; Kofi: β = −0.15, Χ^2^(1) = 2.79, *p* = .095), thus replicating previous findings.

### 3.3 Accuracy

**Fig. 4:**
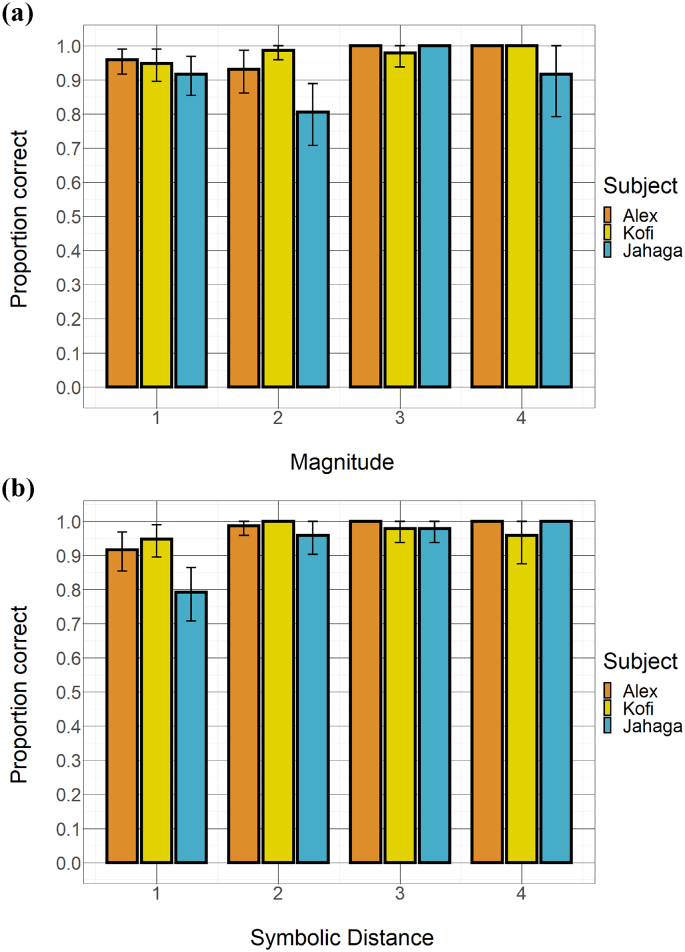
Relationship between (a) magnitude and (b) symbolic distance of subset pairs and chimpanzees' accuracy (proportion correct across trials). Error bars represent confidence intervals (nonparametric bootstrap).

All three subjects were highly accurate in picking the correct item first on the 240 subset trials. Fig. 4a depicts proportion of correct trials as a function of magnitude, Fig. 4b depicts it as a function of symbolic distance. The proportion of correct trials across magnitude categories ranged from 0.93 to 1.00 for Alex, from 0.81 to 1.00 for Jahaga, and from 0.95 to 1.00 for Kofi; and differences in subset magnitude hardly accounted for differences in accuracy (Alex: β = 1.36, Χ^2^(1) = 1.45, *p* = .229; Jahaga: β = 0.26, Χ^2^(1) = 0.17, *p* = .680; Kofi (logistic regression without random intercept for pair): β = 0.80, Χ^2^(1) = 2.82, *p* = .093). For different distance categories, the proportion of correct trials ranged from 0.92 to 1.00 for Alex, from 0.79 to 1.00 for Jahaga, and from 0.95 to 1.00 for Kofi. Likelihood ratio tests revealed these subtle differences to be statistically significant for two subjects (Alex: β = 2.17, Χ^2^(1) = 3.91, *p* = .048; Jahaga: β = 1.49, Χ^2^(1) = 5.30, *p* = .021; Kofi: β = 0.31, Χ^2^(1) = 0.47, *p* = .492), an effect that presumably was carried largely by slightly poorer performance in trials where the two subset items had a symbolic distance of one. A full breakdown of accuracy per subject per item pair can be found in Fig. S4a and S4b (Supplementary Materials).

## 4 Discussion

We presented three chimpanzees with a transitive inference task that probed their responses to pairs of images from a learned list. When choices were more difficult, the chimpanzees also wavered more often. We will discuss, in turn, two conclusions that may be drawn from this. The first is theoretical: because for humans, uncertainty behaviors are correlated with objective task difficulty and subjective experiences of difficulty (Dotan et al., 2018; Questienne et al., 2018; Wokke et al., 2020), our finding provides indirect support for the hypothesis that chimpanzees subjectively experience feelings of uncertainty in similar ways. The second conclusion concerns measurement. Subjects were highly proficient across subset trials from different magnitude and difference categories, and differences in accuracy, where they existed, were very subtle. In spite of these ceiling effects, the distribution of wavering across conditions replicated closely the magnitude and symbolic distance effects that have often been documented for accuracy and response latency in transitive inference tasks with nonhuman primates (Jensen, 2017; Terrace, 2012).^4^ This suggests that wavering could be useful as a highly sensitive, overtly observable measure of response competition in those domains where it plays an important role in theory building, including metacognition (Hampton, 2009; Proust, 2012) and other forms of executive control (Völter, Tinklenberg, Call, & Seed, 2018).

In this study, we addressed the question whether the behaviors shown in response to different levels of difficulty are similar in humans and chimpanzees to make the case that the emotional experience of uncertainty is similar across species, as has been suggested by some (e.g. Couchman, Beran, Coutinho, Boomer, & Smith, 2012). As for any other study of animal emotion, we acknowledge that a single study that shows a task-behavior correspondence across two closely related species cannot solve the question of subjective experience. Rather, studying emotions in nonhuman animals requires a componential approach (Mendl et al., 2010; Panksepp, 2010; see also Carruthers, 2017). Evidence that across species, specific patterns of (neuro-)physiological activation (e.g. neural vacillation, Kaufman, Churchland, Ryu, & Shenoy, 2015; EEG signatures, Bosc et al., 2017; thermal imaging, Kano, Hirata, Deschner, Behringer, & Call, 2016) and cognitive responses (e.g. improved memory for trials with pronounced uncertainty behavior) also correlate reliably with differences in task difficulty, as well as with wavering and other potential indicators of uncertainty like scratching (cf. Call, 2012), would further strengthen our case. Beyond anthropocentric triangulation of what uncertainty might “feel like” for an animal, the componential approach helps in discerning which combinations of situations and response profiles are reliably distinct from one another. This is key to exploring the emotional diversity in a given species (Paul et al., 2005). Distinct emotional response profiles are often argued to represent adaptations to specific selection pressures, adaptations that may support fast motor responses (e.g. Lang, Davis, & Öhman, 2000), navigation of social relationships (Waller & Micheletta, 2013) or learning from experience (Baumeister, DeWall, Vohs, & Alquist, 2010). The same may be true for distinct epistemic emotions that animals may experience, e.g. feelings of familiarity vs. feelings of certainty may be involved in different adaptive response profiles.

In humans, uncertainty behaviors correlate not only with reported feelings of uncertainty but also with explicit metacognitive judgments (Dotan et al., 2018; Questienne et al., 2018). As in the study by Dotan et al. (2018), we found that gradual increases in task difficulty corresponded to gradual increases in response competition in the form of wavering. Speculation about whether our chimpanzees' episodes of wavering were also accompanied or followed by metacognitive judgments of this sort would be premature, as our task did not create opportunities for second-order behaviors like information seeking, opting out, or wagering. Rather, the chimpanzees' wavering behaviors can be regarded as manifestations of the cognitive conflict that is assumed to be at the beginning of many metacognitive processes (Beran et al., 2012; Smith et al., 1995).

As humans appear to rely quite often on metacognitive heuristics that exploit self-generated motor behavior – be they implicit or explicit^5^ – we believe it to be likely that nonhuman primates also use, or at least can learn to use, similar heuristics to motivate second-order behaviors (see Hampton, 2009). Future studies of animal metacognition may thus benefit not only from allowing subjects to express wavering and similar behaviors, but from actively encouraging and quantifying these. For example, is hesitation with wavering more often followed by seeking information or opting out than hesitation without wavering? Is metacognitive sensitivity – the correlation between confidence and accuracy – higher in tasks that, by design, create opportunities for wavering than in tasks that do not? This suggestion is not meant to be taken in opposition to efforts to exclude publicly available cues in order to refute associationist explanations (Hampton, 2009; Proust, 2012). Rather, studies that allow response conflict to be expressed should be seen as an additional avenue. In this case, the demonstration that the metacognitive behavior is not a mere result of associative learning, would rest on flexible and targeted responding rather than on what may serve as the eliciting cue (Beran et al., 2013; Bohn et al., 2017; Call, 2010; Krachun & Call, 2009; Marsh, 2019).

In the developmental and educational literature, it is often suggested that for humans, the relationship between first-order, self-generated cues to task difficulty (e.g. response fluency or hesitation) and conflict-resolving second-order behaviors (e.g. self-testing or using mnemonics) are not “instinctive” or “spontaneous”. Rather, many of these introspective strategies need to be learned (Dokic, 2012; Heyes, Bang, Shea, Frith, & Fleming, 2020; Karpicke, Butler, & III, 2009). This may be true for animal metacognition, too, at least in some cases. If animals also use strategies that exploit monitoring of self-generated behavior, then future studies may benefit from looking separately at three elements: (1) the tendency to express cognitive conflict with wavering or other uncertainty behaviors, (2) the general ability to exploit behavioral cues by responding to them e.g. with information seeking, and (3) the ease with which such exploitation strategies can be learned.

For example, each of these three levels could be considered in the study of risk tolerance, which has been suggested as a potential intra- and interspecies moderator of metacognitive responding (Beran, Perdue, Church, & Smith, 2016; Call, 2010; Carruthers, 2017). It could be illuminating to this debate to compare whether it is the first-order uncertainty behaviors that are already more readily expressed in those individuals or species that are considered to be less risk-tolerant than others (e.g. rhesus macaques vs. capuchins, see Beran et al., 2016; or bonobos vs. chimpanzees, see Heilbronner, Rosati, Stevens, Hare, & Hauser, 2008). There is some evidence consistent with this in humans, for example, a recent computer mouse-tracking study with human participants demonstrated a close relationship between tracking metrics that were comparable to the operationalization of wavering used in our study and subjective risk perception as well as individual risk aversion (Stillman, Krajbich, & Ferguson, 2020). Alternatively, risk-averse individuals or species may differ more strongly with regard to how sensitive they are in noticing their self-generated cues, or in learning to respond to them with second-order behavior. Another domain in which studying uncertainty behaviors could be very beneficial is the relationship between metacognition and theory of mind that is often at the heart of discussions of the evolution of either (Carruthers, 2009). Humans are not only good at exploiting their own self-generated motor responses for metacognitive judgments, they can also use subtle motor behavior expressed by a competitor to predict what they are about to do (Vaziri-Pashkam, Cormiea, & Nakayama, 2017). This raises the question, for humans and other primate species alike, whether individual tendencies to exploit self-generated motor behavior are associated with higher accuracy in predicting others' future actions as well.

There are a number of limitations to this investigation. First, as described in the Methods section, serial learning training was not completed with all chimpanzees with whom it was attempted, and thus some subjects that may have eventually succeeded did not participate in the test. Selection bias of this type would introduce problems to the interpretation of individual differences across tasks (e.g. regarding the relationship between wavering and risk aversion, executive functions or theory of mind, as proposed above, see e.g., Morton, Lee, & Buchanan-Smith, 2013). Future studies may thus seek to study wavering under uncertainty in tasks that are easier to acquire and thus reduce selection bias, e.g. simple “sparse vs. dense” discrimination tasks as they have long been used in animal metacognition research. Second, as discussed in the Methods section, our task did not control systematically for the spatial distance between stimuli across different levels of difficulty. Though, due to complete randomization of spatial distances, this did not turn out to be a confound in this study, future studies may more proactively control the effect of spatial distance by keeping it constant, or by varying it across conditions in a completely counterbalanced manner to ensure that wavering always remains equally detectable.

Finally, regarding wavering in the specific context of research on transitive inference, it may be regarded as a limitation that our test design did not cleanly separate the effects of magnitude and distance from potential confounds that are often given special consideration in research on serial learning and inference. These confounds resulted primarily from the fact that testing time constraints only allowed us to train our subjects in completing a comparatively short list (five items). For example, to maximize the number of distance categories available for analysis, we included the largest distance category, which was represented by only a single pair of items (“1–5”). This pair included the first and the last item of the learned list, and so “terminal item effects” (Jensen, 2017) may have contributed, beyond symbolic distance, to this category being less difficult than others. Similarly, different difficulty categories were represented in this study by different numbers of item pairs (e.g. magnitude category “1” is represented by four pairs while magnitude category “4” is represented by only one pair). Future studies that seek to relate wavering behaviors to, e.g. the uncertainty of an item's position as it is estimated in computational models of list learning (Jensen et al., 2015) may take full advantage of the methods of statistical control that have been developed in this field (e.g. using longer lists, using multiple lists, excluding item pairs from analysis that include the first or last list item).

In conclusion, our results show that subtle behavioral cues of uncertainty can be measured non-invasively in nonhuman primates. In close analogy to humans, the extent to which subjects wavered was closely related to objective task difficulty and revealed subtle differences in proficiency in a task in which subjects were otherwise highly accurate across conditions. We suggest that studies in the field of animal metacognition routinely incorporate measurements of uncertainty behaviors to inform debates of epistemic emotions and procedural metacognition.

## Credit author statement

Conceptualization & Methodology: MA, JC, EM; Investigation: MA, EM; Data curation: MA, EM; Software: MA; Formal analysis: MA, EM, JC; Visualization: MA, EM; Writing - original draft: MA, EM, JC; Writing - review & editing: MA, EM, JC; Resources, Funding Acquisition & Supervision: JC.

## Appendix A Supplementary data

Supplementary video SV1 Wavering hand movements by chimpanzees Supplementary video SV1

Supplementary material Image 1

## Footnotes

1 Response competition behaviors have been referred to as “hesitation” (Muenziger, 1938), “ancillary motor behaviors” (Smith et al., 1995), “vacillation” (Hampton, 2009), “oscillation” (Proust, 2012) or simply “wavering” (Sayers et al., 2015; Smith et al., 1995). In the field of rodent navigation, looking back and forth between maze alley options, has been termed “vicarious trial and error”, and has, based on neuroscientific investigations, been linked to deliberative mental simulations of future actions (Redish, 2016). If and when other behaviors like hand wavering or gaze alternations also imply serial representations of hypothetical outcomes has yet to be determined. Here we will use the term “uncertainty behaviors” for the general class of behaviors that have been described with these different terms, and “wavering” specifically for behaviors that involve movement back and forth between multiple physical options or hesitating in proximity to one of them.

2 We recognize this as an indirect argument: if human verbal reports of specific subjective experiences (e.g. feeling uncertain) in specific situations (e.g. a more difficult decision) are accompanied by specific behaviors (e.g. visibly wavering between options), then observing comparable behaviors in comparable situations in nonhuman animals lends plausibility to the idea that the animal's subjective experience is also comparable. For example, Couchman et al. (2012, p.32) explicitly make this argument, speculating about the “experience of uncertainty-monitoring” in primates. Similar indirect arguments for animal subjective experience are central in other areas of comparative psychology, e.g. the study of basic emotions and their feeling components (Mendl, Mason, & Paul, 2017; Panksepp, 2010, Panksepp, 2011) or the study of episodic memory and its autonoetic components (Dere, Kart-Teke, Huston, & De Souza Silva, 2006; Tulving, 2005). Whether such an indirect argument is permissible in any domain is one of comparative psychology's eternal debates and will not be settled here (see Wemelsfelder, 1997, for a discussion).

3 In reference to the computer task used in this study, we use a broad definition of the term “transitive inference task” as it is endorsed by e.g. Jensen et al. (2013). A stricter definition may only recognize a task as a “pure” test of transitive inference when the test is preceded only by training individual premise pairs (paired associates training). This is because only the paired associates training method ensures that subjects cannot learn associatively, e.g. about the relationship between “B” and “D” from the simultaneous presentation of these items (as is possible in serial or simultaneous chaining training). Put differently, calling our task a transitive inference task serves to describe the commonality in the testing method, without weighing in on the question of whether inferential vs. associative accounts better explain performance.

4 It is not unusual in transitive inference and serial learning studies that item position effects are manifest primarily in accuracy or latency, but not both (Templer et al., 2019). The finding in this study that item position effects were found for wavering (and latency), but were absent or very subtle for accuracy, is consistent with this.

5 Questienne et al. (2018) use causal language that suggests that exploitation occurs (“resulting in”, “determined by”), but remain non-committal as to whether it is metarepresentational: “Whether this relationship results from an explicit strategy (i.e.’I was slow, therefore I report stronger urge-to-err’) can be debated.” (ibid.). This mirrors notes of caution about animal behavior that just because animals may exploit cues of decisional conflict (even purely endogenous ones), this is not sufficient evidence that the cognitive control process explicitly represents this exploitation, a feature that some require to be fulfilled to speak of “meta”-cognition (Carruthers, 2014).
