# Supplementary material for: Chimpanzees (Pan troglodytes) show subtle signs of uncertainty when choices are more difficult
Source: Cognition. 2021 Sep;214:104766. doi: 10.1016/j.cognition.2021.104766 (PMC8346948; doi:10.1016/j.cognition.2021.104766)
Supplement: Supplementary file 2 — Supplementary material [file mmc2.pdf]

Chimpanzees (*Pan troglodytes*) show subtle signs of uncertainty when choices are more difficult

Matthias Allritz, Emma Suvi McEwen, Josep Call

Supplementary Materials

Fig. S1: Training progress across sessions

Fig. S2: Wavering movements for individual item pairs

Fig. S3: Latency for individual item pairs

Fig. S4: Accuracy for individual item pairs

Fig. S5: Comparison of model predictions and wavering data

Fig. S6: Wavering across trials

Fig. S7: Latency across trials

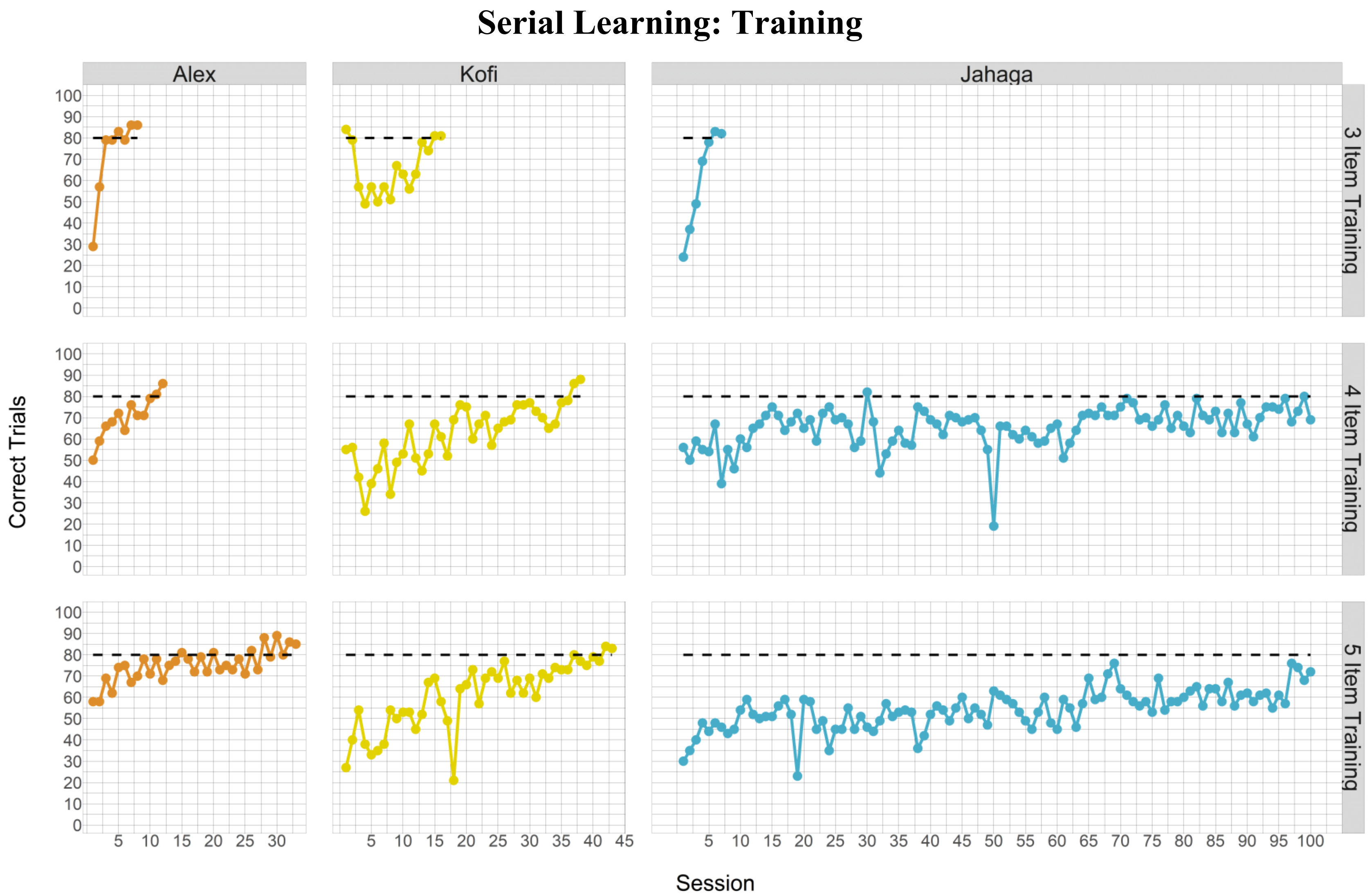

Fig. S1. Training progress across sessions for each subject. Subjects were promoted to the next training stage, or to test, when they had completed two consecutive sessions with performance above criterion (dotted line), or when they had completed a total of 100 training sessions.

Descriptive statistics for individual item pairs

Figures S2-S4 depict wavering, response latency, and mean accuracy (proportion correct) for individual item pairs. In each case, the left figure presents pairs in clusters that represent the same magnitude category, whereas the right figure presents pairs in clusters that represent the same distance category. In descriptive terms, magnitude and distance effects interacted in various ways for the three chimpanzees such that, e.g., a magnitude effect, when holding distance constant, may be apparent only for one or two distance categories for a specific subject and dependent measure.

Wavering movements for individual item pairs

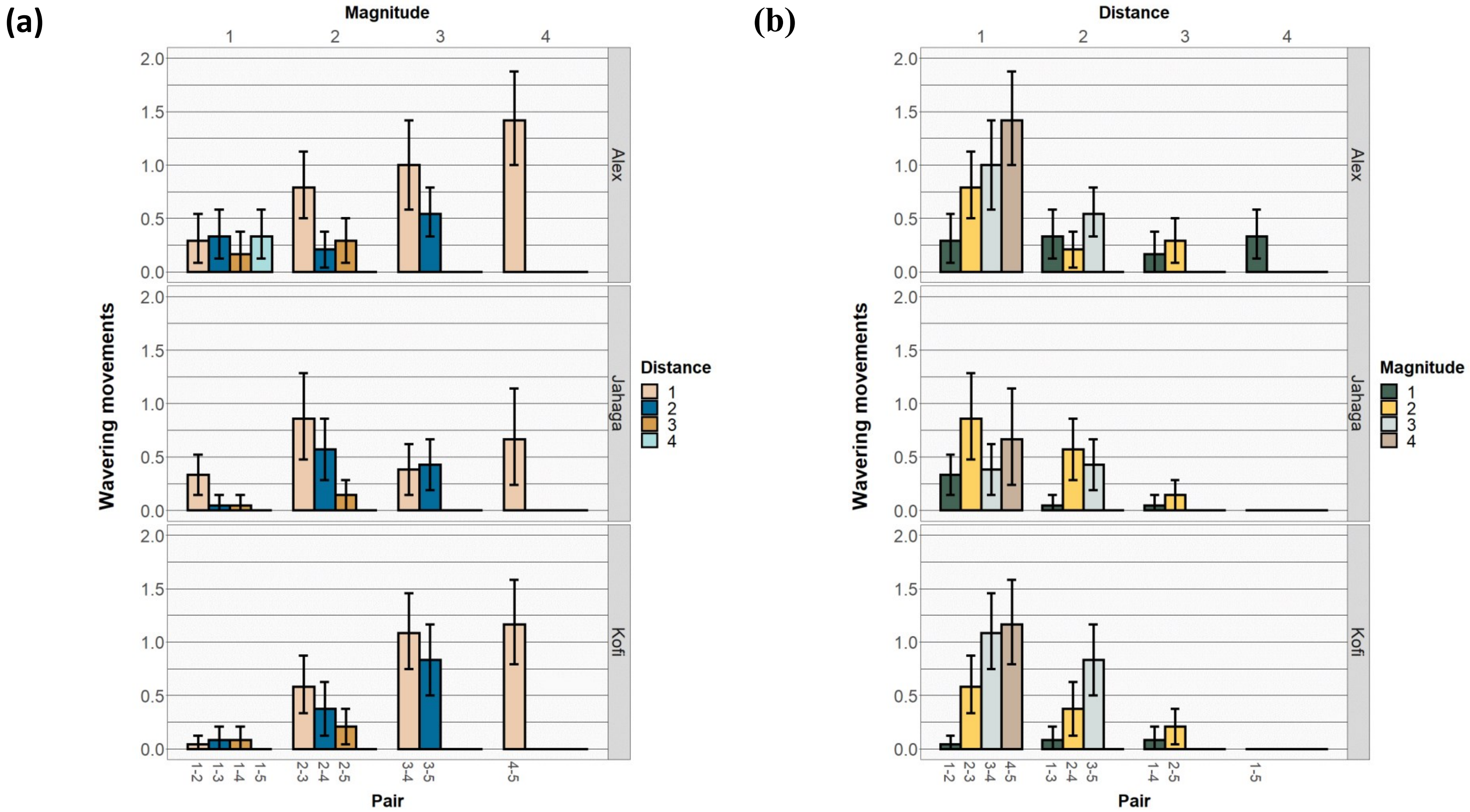

Fig. S2. Mean number of wavering movements for individual item pairs, with item pairs sorted by magnitude (a), or distance (b). Both graphs depict the same information. Error bars represent confidence intervals (nonparametric bootstrap).

Latency for individual item pairs

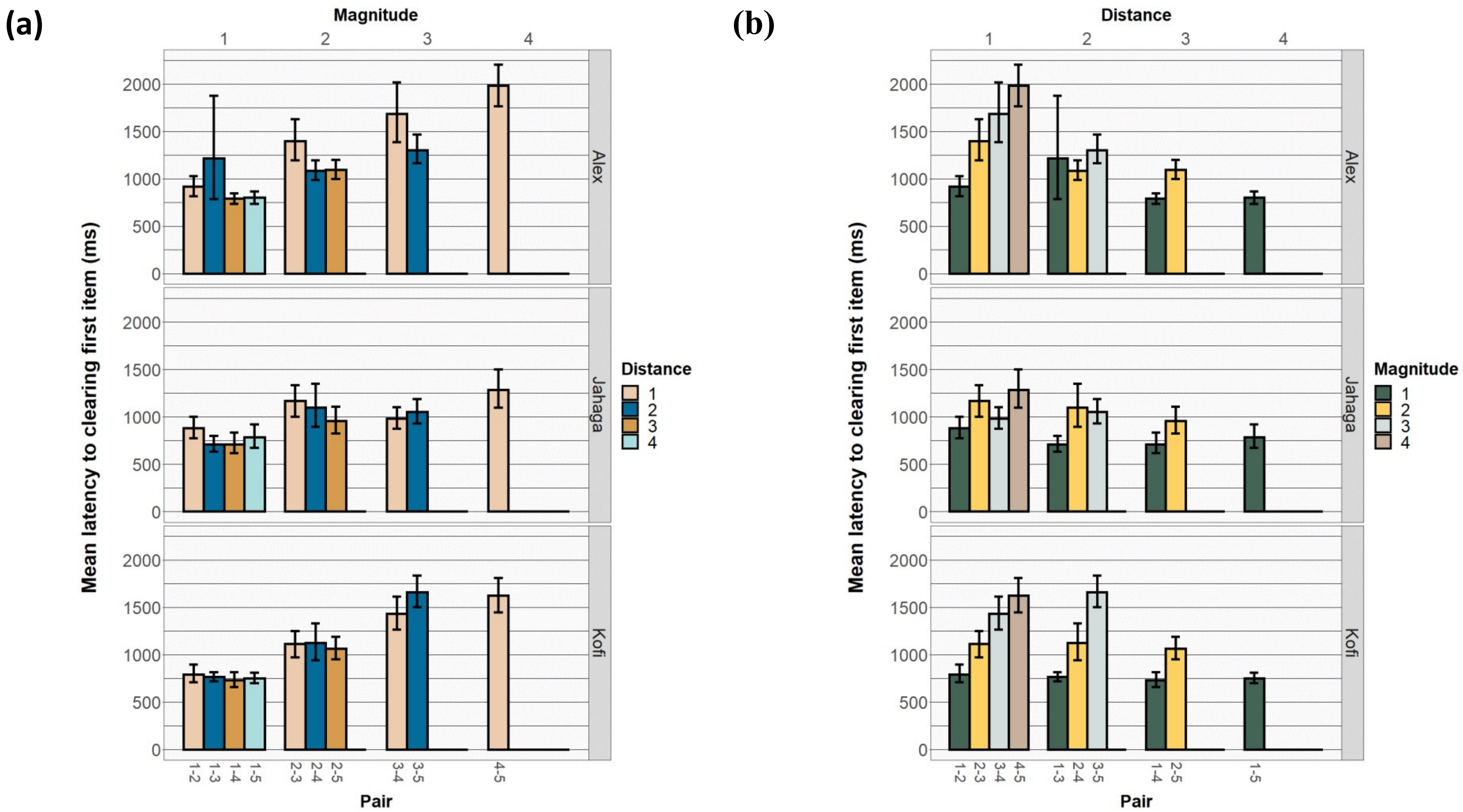

Fig. S3. Latency for individual item pairs, with item pairs sorted by magnitude (a), or distance (b). Both graphs depict the same information. Error bars represent confidence intervals (nonparametric bootstrap).

Accuracy for individual item pairs

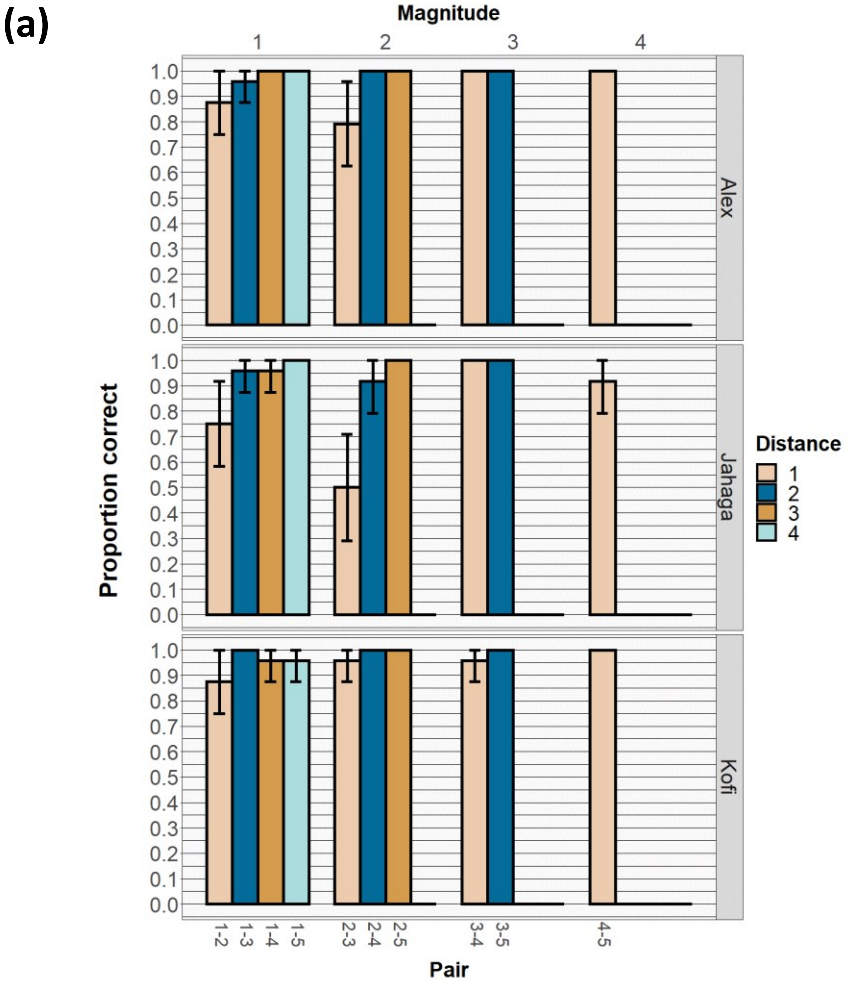

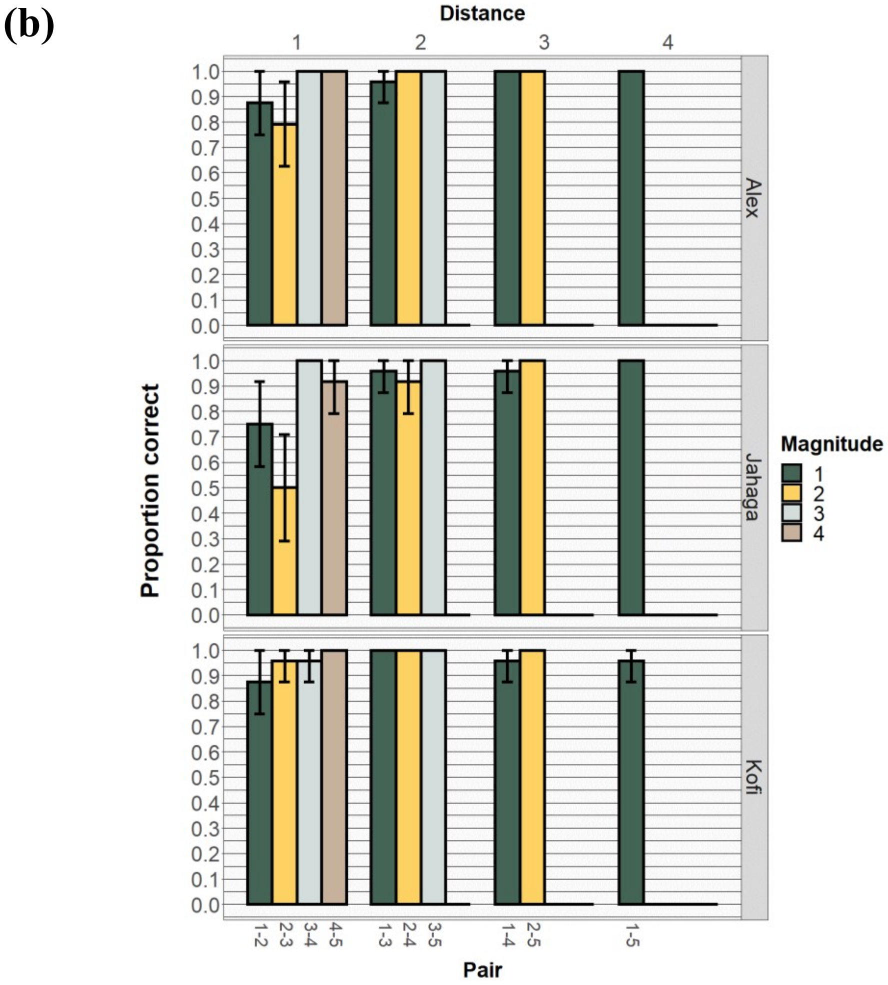

Fig. S4. Accuracy for individual item pairs, with item pairs sorted by magnitude (a), or distance (b). Both graphs depict the same information. Error bars represent confidence intervals (nonparametric bootstrap).

Comparison of wavering model predictions and empirical data

(a)

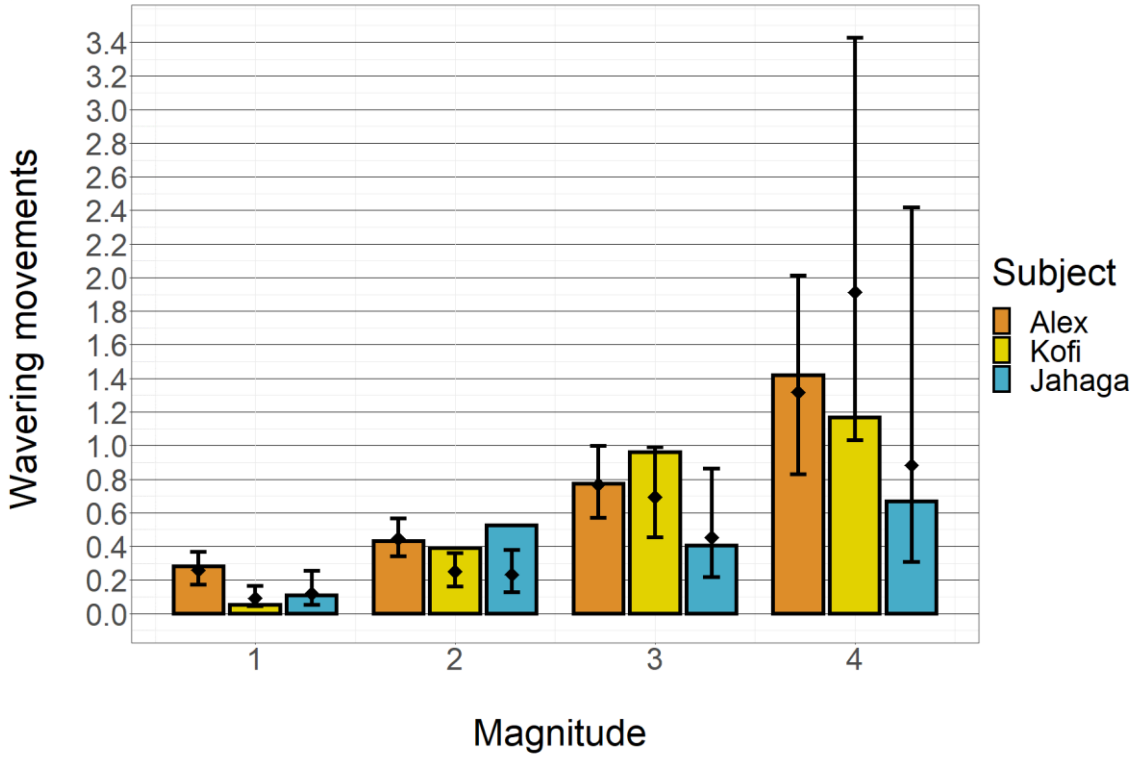

(b)

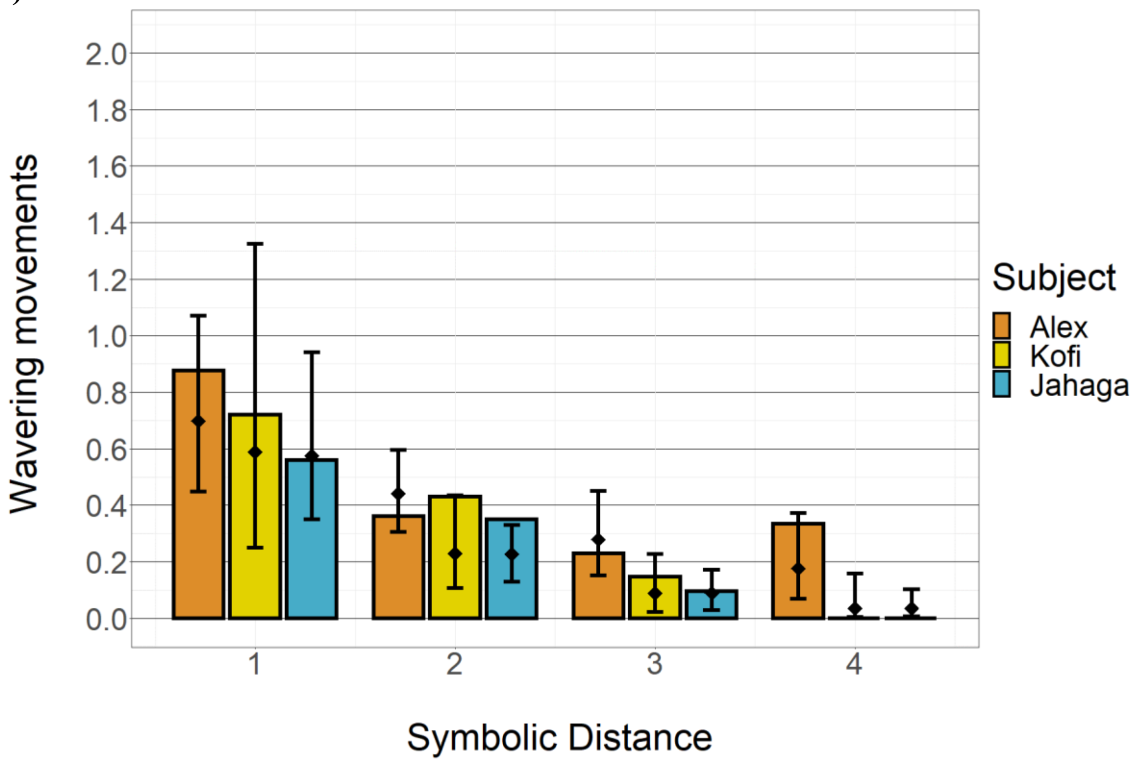

Fig. S5. Comparison of Poisson model predictions and wavering data for (a) magnitude and (b) symbolic distance categories. Colored bars represent sample means of wavering behaviors, diamonds represent point predictions (fitted values via fixed effects model formula, “trial” value set to mean), error bars represent 95% confidence intervals around point predictions obtained via 1000 parametric bootstraps (lme4 function bootMer, argument “use.u” set to “true”).

Figures S5a and S5b contrast point predictions based on fitted models (black diamonds and error bars) with the empirical wavering data (colored bars). As can be seen in Fig. S5b, distance models appeared to capture the structure of the empirical data slightly better than magnitude models, which overestimate the amount of wavering for the largest magnitude category for two subjects (Jahaga and Kofi), while underestimating wavering for magnitude category "2".

Trial effects

In an exploratory analysis, we tested for the significance of the effect of trial, testing whether individuals' frequency of wavering, their response latency, or their accuracy increased or decreased across trials of the same magnitude or distance category. To minimize alpha error inflation, in line with the significance testing philosophy endorsed by Forstmeier and Schielzeth (2011), in each case we first conducted a full-null model comparison, comparing a model with both predictors (e.g. accuracy and trial) against a model that only included the random effects terms. Only if this comparison proved significant by conventional standards ($p < .05$), did we compute a p-value for the trial effect itself by comparing the full model against a reduced model that included the other main effect (e.g. magnitude) and the random intercept term, but not the effect of trial.

Wavering. For models of wavering as a function of magnitude, full-null model comparisons showed a significant difference for all three subjects (Alex: $X^2(1) = 11.85$, $p = .003$; Jahaga: $X^2(1) = 9.43$, $p = .009$; Kofi: $X^2(1) = 25.70$, $p < .001$). For two of the subjects, Jahaga and Kofi, there was a significant effect of trial, such that in later trials wavering became more rare (Kofi: $X^2(1) = 9.14$, $p = .003$; Jahaga: $X^2(1) = 3.95$, $p = .047$; Alex: $X^2(1) = 0.63$, $p = .427$). For models of wavering as a function of distance, full-null model comparisons showed a significant difference for two subjects, Jahaga and Kofi (Jahaga: $X^2(1) = 14.96$, $p = .001$; Kofi: $X^2(1) = 14.85$, $p = .001$; Alex: $X^2(1) = 5.99$, $p = .050$). For Jahaga and Kofi, the trial effect estimates and results of likelihood ratio tests were nearly identical to the ones obtained from the models that included magnitude as predictor, such that wavering became rarer across trials (Kofi: $X^2(1) = 9.14$, $p = .003$; Jahaga: $X^2(1) = 3.95$, $p = .047$, see also Fig. S6).

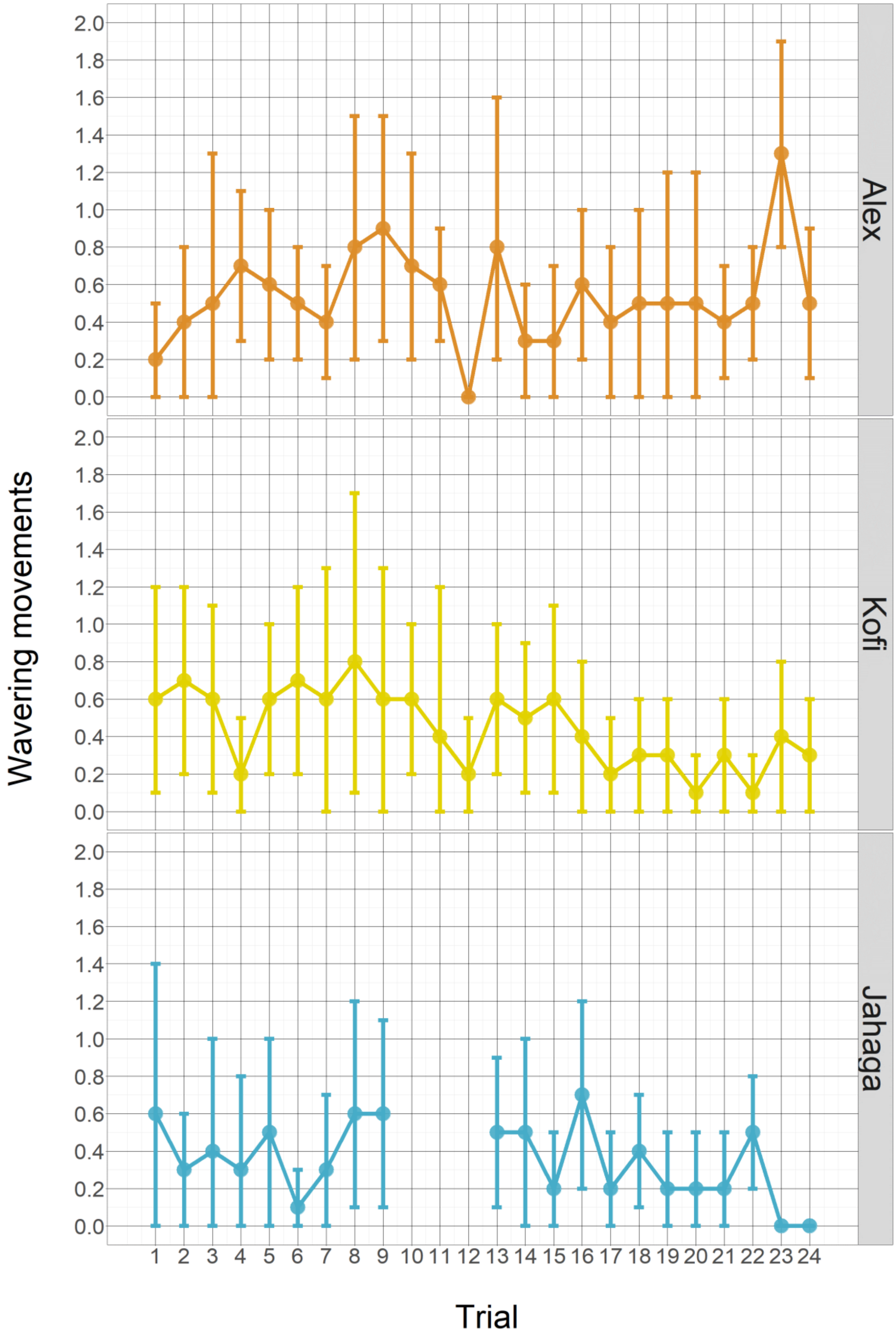

Fig. S6. Wavering across trials. Each point represents the mean of wavering counts across the10 item pairs. Error bars represent confidence intervals (nonparametric bootstrap). Note that for subject Jahaga, Session 4 was not available for behavior coding (see main article).

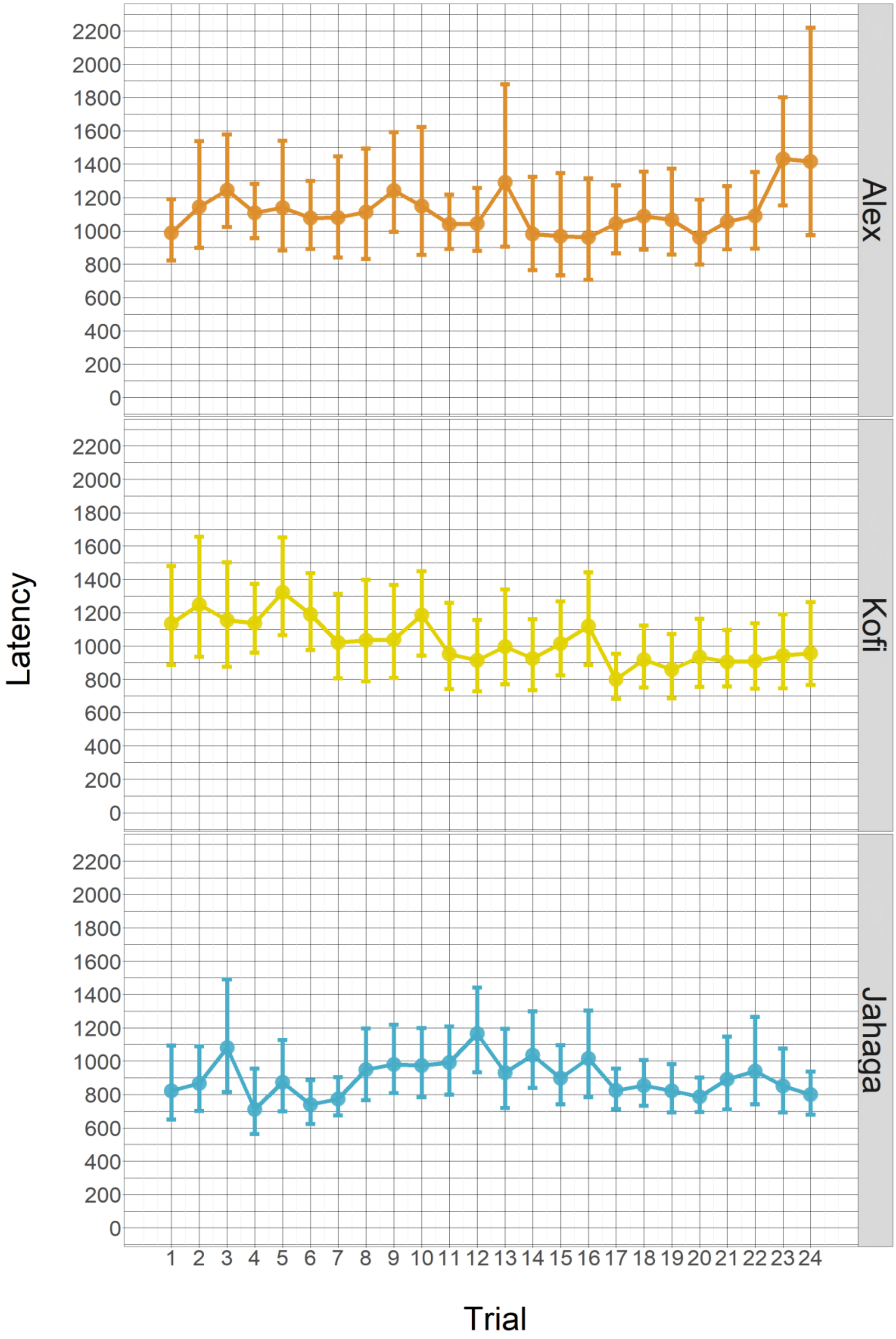

Fig. S7. Latency to touch first item across trials. Each point represents the mean latency across the 10 item pairs. Error bars represent confidence intervals (nonparametric bootstrap, log-transformed values converted back to original scale).

Latency. For models of latency as a function of magnitude, full-null model comparisons showed a significant difference for all three subjects (Alex: $X^2(1) = 23.06$, $p < .001$; Jahaga: $X^2(1) = 12.49$, $p = .002$; Kofi: $X^2(1) = 58.11$, $p < .001$). For one subject (Kofi), there was a significant effect of trial, such that in later trials the subject responded faster (Kofi: $X^2(1) = 31.07$, $p < .001$; Alex: $X^2(1) = 0.29$, $p = .587$; Jahaga: $X^2(1) < 0.01$, $p = .947$). For models of latency as a function of distance, full-null model comparisons showed a significant difference for two subjects (Alex: $X^2(1) = 6.49$, $p < .039$; Kofi: $X^2(1) = 33.86$, $p < .001$; Jahaga: $X^2(1) = 4.71$, $p = .095$). The individual effect of trial was significant only for Kofi, reflecting the same trend of becoming faster across trials (Kofi: $X^2(1) = 31.07$, $p < .001$; Alex: $X^2(1) = 0.29$, $p = .587$, see also Fig S7).

Accuracy. For models of accuracy as a function of magnitude, we fitted GLMMs for Alex and Jahaga, and a GLM for Kofi (see main article). For Alex and Jahaga, LRTs of full-null model comparisons did not show a significant difference, reflecting the ceiling effect of accuracy ($X^2(1) = 3.72$, $p = .155$, and $X^2(1) = 0.67$, $p = .761$, respectively). For Kofi, comparing a model that included trial and magnitude with a model that included only magnitude also did not reveal a significant effect of trial ($X^2(1) = 1.47$, $p = .226$). For distance models (all GLMMs), full-null model comparisons did not show a significant difference for Jahaga ($X^2(1) = 5.80$, $p = .055$) or Kofi ($X^2(1) = 1.94$, $p = .380$). The full-null model comparison for Alex was significant ($X^2(1) = 6.19$, $p = .045$), however, the individual effect of trial was not ($X^2(1) = 2.24$, $p = .134$).

Wavering: coding scheme

Note: For all analyses reported in the main article, including the analysis of interobserver reliability, “Turns”, “Turns*”, and “Rests” were collapsed into a single category of “Wavering”.

Turn:
A single frame. The subject’s finger or knuckle changes its direction of movement *while* above or *while on the way* to a stimulus. *While on the way* means that

1. It has to be clear without confusion which stimulus the hand movement was directed to
2. The finger must have *reached* at least *the critical area:*

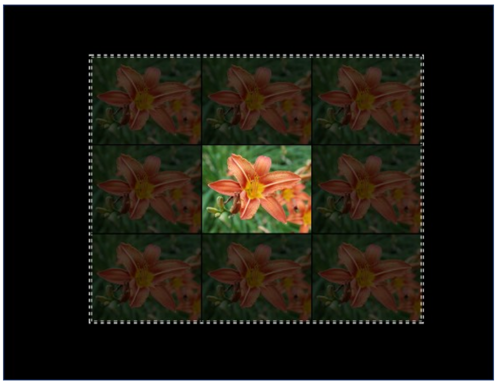

The *critical area* is defined as the area that is covered by the stimulus and the area surrounding it with a maximum distance equal to the size of another stimulus.
Reaching means that the position of the fingertip at the apex of its movement would fall within the *critical area* if the screen was touched by the subject at this moment. Try to take video camera angle and the subject’s perspective into account.

Do not code Turn if the subject’s finger or knuckle passes a stimulus “on its way” to another stimulus without an apparent change of direction of movement. A Turn code always marks the end of a Move code.

If after a “Turn” the subject’s finger returns to the same stimulus / critical region that justified that Turn code, without approaching another critical area closely enough in between, code this as **Turn***.

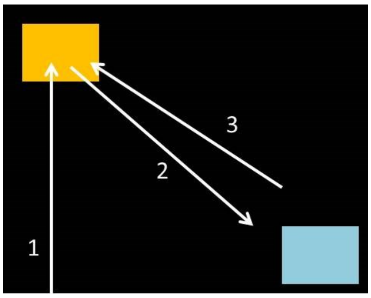

two regular Turns

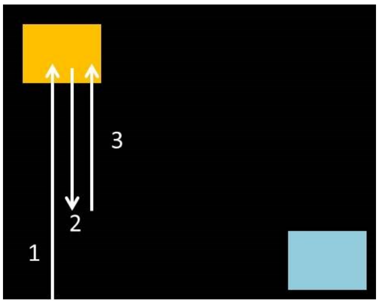

Turn*

Rest:
Duration code. The subject’s finger hovers directly over or in very close proximity to a stimulus without touching it. Resting can be done with tip of finger, thumb or knuckle (hereto forth called finger). Code Rest when subject's finger stays for at least *three consecutive frames* (= 0.12s) within the vicinity of the stimulus, either ‘hovering’ or not moving at all. *Hovering* means that the finger is moving in a way that does not look like it is part of the trajectory toward touching any stimulus. Code Rest as soon as the finger has come to rest clearly above a stimulus (same frame as the end of the preceding Move or Retreat Arm code) and rests there according to the above description. A Rest Code is always followed by a Touch, Retreat Arm, or Move code (not by a Turn code!)

Touch:
A single frame. The subject touches the screen at the position of a stimulus (or very close to it). Touching can be done with tip of finger, thumb or knuckle. Typically the coded touching event coincides with the stimulus disappearing. If a stimulus has clearly been touched but it is not registered (the stimulus does not disappear), code **Touch (not registered).** Note that any Touch code, registered or not, should be followed by a Move code (either to a different stimulus, or to the same), or a Retreat Arm code.

Move to:
Duration code. The subject moves their hand *from* a position that justifies a Touch, Turn or Rest code associated with any stimulus *to* the next position that justifies a Touch, Turn or Rest code associated with any stimulus. *Beginning and End of Move*: when hand motion begins / ends, i.e. the first frame in which the finger, knuckle etc. actually moves away from previous touching, resting, or turning position / arrives at next touching, resting, or turning position. If the hand movement from one stimulus to another is not direct (e.g. if there is a slight side movement to another position on the screen that is not approached closely enough to justify a Touch, Rest, or Turn code), ignore this and code as if it was one continuous move. Sometimes it is difficult to assess whether a stimulus, or which

stimulus, was approached “closely enough” because it is located at a position on the screen where it is not visible to the coder. If from other movements and the outcome of the trial it can be deduced which one it was, code accordingly. If it cannot be deduced, code nothing.

Retreat arm:
Duration code. The subject retreats hand away from screen (more than one hand’s length = from wrist to tip of index finger) for more than 1 second (25 frames), begin code as subject begins to retreat hand, end “Retreat arm” code with next Touch, Turn or Rest code.

Retreat body:
Duration code. The subject leaves the touch screen.

Beginning of Trial:
A single frame. Initiation symbol appears on screen.

Beginning of Stimulus Presentation:
A single frame. The stimuli appear on screen.

End of Trial:
A single frame. All stimuli have completely disappeared from screen.

References

Forstmeier, W., & Schielzeth, H. (2011). Cryptic multiple hypotheses testing in linear models: Overestimated effect sizes and the winner’s curse. *Behavioral Ecology and Sociobiology*, *65*(1), 47–55. https://doi.org/10.1007/s00265-010-1038-5
